# *Rhodococcus equi*, an Unusual Human Pathogen That Causes Cavitating Pneumonia in Patients With AIDS

**DOI:** 10.1155/2024/5570186

**Published:** 2024-11-07

**Authors:** J. Vandersnickt, Pee J. Van, S. Vandamme, C. Kenyon, E. Florence, S. Van Ierssel, E. Vlieghe, C. Theunissen

**Affiliations:** ^1^Department of General Internal Medicine, Infectious Diseases and Tropical Medicine, University Hospital Antwerp, Antwerp, Belgium; ^2^Department of Microbiology, University Hospital Antwerp, Antwerp, Belgium; ^3^Department of Clinical Sciences, Institute of Tropical Medicine, Antwerp, Belgium

**Keywords:** HIV, immune suppression, pneumonia, *Rhodococcus equi*

## Abstract

*Rhodococcus equi* is a rare human opportunistic pathogen that has been increasingly reported in recent decades. It mainly affects immunosuppressed patients, and in particular human immunodeficiency virus (HIV)–infected patients, where it typically presents as cavitary pneumonia. Early treatment with combined and effective antimicrobials and antiretroviral therapy after prompt diagnosis is essential to ensure an optimal outcome. We present a case series of three human *Rhodococcus equi* infections in HIV-infected patients with advanced immune suppression, treated at the University Hospital of Antwerp, Belgium.

**Summary**

• *R. equi* should be considered in the diagnosis of cavitary pneumonia, particularly in immunosuppressed patients.
• Close collaboration with the microbiology department is essential to optimize diagnosis and treatment and determine antimicrobial drug susceptibility.
• Treatment should be tailored to the clinical presentation and underlying immune suppression and contain a combination of active antibiotics.
• Treatment should include restoring the immune status of patients.
• Currently, primary or secondary prophylaxis does not play a role, but patients at risk (immunocompromised with exposure to farming/livestock environment) should be adequately advised.
• The need for isolation measures is still unclear, since a few cases of nosocomial and person-to-person infections have been described.

## 1. Introduction

Originally, *Rhodococcus equi* was isolated from the lungs in foals and identified as *Corynebacterium equi* (1923) [1]. Subsequently, its characteristics were found to be more closely related to mycobacteria and *Nocardia* spp., which was the reason for its reclassification as *Rhodococcus* (“red-pigmented coccus”) [2]. It is a multihost Gram-positive pleomorphic coccobacillus found in the intestinal tract and the feces of many herbivores but is also isolated from porcine species, birds, water animals, and cats and dogs for example [3, 4]. *R. equi* is a rare opportunistic pathogen, and the first human case dates from 1967 [5]. Reporting can be underestimated because of misclassification [4].

*R. equi* is an intracellular pathogen that infects many cell types but is best replicated in macrophages and causes typical cavitating necrotizing pyogranulomatous lesions, especially in humans with decreased cell-mediated immunity [4]. Most of the reported/published cases are patients infected with human immunodeficiency virus (HIV) before the era of highly active antiretroviral therapy (HAART) [6]. *R. equi* typically presents with symptoms of pulmonary involvement. Treatment is challenging and often requires a long combination therapy with different active antimicrobials [7]. The advent of HAART has improved the long-term prognosis of HIV-associated *R. equi* infections [7].

In this case series, we describe three patients with *R. equi* infection treated at the University Hospital of Antwerp, Belgium.

## 2. Case Presentations

### 2.1. Case 1

A 37-year-old man, diagnosed with HIV in 2006, presented at the emergency unit in December 2010 with hemoptysis and poor general condition. HAART had been prescribed, but poor therapy compliance resulted in immunological and virological failure with a CD4 count of 13 cells/mm^3^ (1.9%) and a HIV viral load of 18.800/*μ*L. He had been treated for tuberculosis (TB) in the past with an unknown regimen.

**Figure 1:**
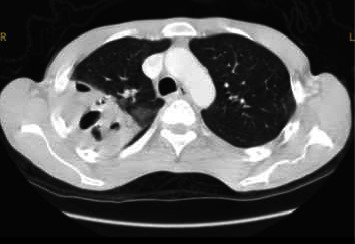
Chest computed tomography: segmental necrotizing pneumonia with cavitation of the right upper lobe.

Physical examination presented a cachectic man with normal parameters but diminished breath sounds in the right upper lobe. Lab tests did not show leukocytosis and a low level of C-reactive protein (CRP) of 9.5 mg/dL (< 0.30 mg/dL). A chest CT showed a large cavitating lesion in the right upper lobe (Figure 1). Ziehl–Neelsen staining and qPCR in three sputum samples and bronchoalveolar lavage (BAL) fluid were negative for *Mycobacterium tuberculosis*. Direct microscopy showed a purulent bronchus aspirate and BAL, and Gram staining showed Gram-positive cocci and culture *Rhodococcus* species (> 10^5^ CFU/mL). *R. equi* was confirmed by analytical profile index (API) identification in both samples. No data on susceptibility testing could be retrieved. Dual therapy for *Rhodococcus* was started with clarithromycin (500 mg twice a day) and rifabutin (150 mg three times a week), and because of a high clinical suspicion of TB, an adapted regimen with ethambutol (1200 mg once a day), isoniazid (300 mg once a day), and pyrazinamide (1500 mg once a day) was added to the antirhodococcal treatment.

One month after starting treatment, the patient showed clinical deterioration with increased cough and hemoptysis. The physical examination and laboratory results were unchanged. Mycobacterial cultures of sputa and BAL fluid were negative. A chest radiograph showed a growth of the right upper lobe lesion. Oral *Rhodococcus* treatment was considered to have failed and intravenous meropenem was added to his treatment for 2 weeks. Subsequent clinical evolution was favorable. Additional qPCR testing on initial sputum returned positive after a few weeks for non-tuberculous mycobacteria, without species determination despite sequencing. Treatment was simplified to clarithromycin, rifabutin, and ethambutol, covering both suspected *Mycobacterium avium* complex and *Rhodococcus* spp. and administered for a total of 6 months with complete clinical cure.

### 2.2. Case 2

A 46-year-old woman came to the emergency department in September 2010 with progressive shortness of breath, accompanied by high-grade fever and a productive cough for one month. She had been diagnosed with HIV and advanced immunosuppression in 1994. Adherence to antiretroviral therapy was poor, and she stopped taking HAART one month before admission. The medical history included nevirapine-induced Stevens–Johnson syndrome and allergies to penicillin, sulfonamides, and quinolones. The patient lived on a farm with goats but reported no other animal contact. She had no relevant travel history.

**Figure 2:**
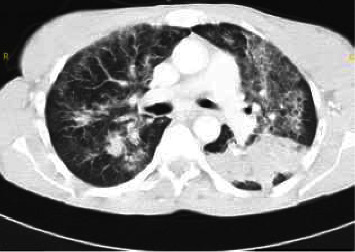
Chest computed tomography of Case 2: diffuse bilateral infiltrates mainly alveolar.

Clinical examination revealed a body temperature of 40.5°C, an oxygen saturation of 88% while breathing room air, and central cyanosis. Lung auscultation revealed bilaterally decreased breathing sounds with diffuse crepitations. Laboratory studies showed a total leukocyte count of 10.5 × 10^9^/L and a CRP level of 15 mg/dL, CD4 count was 24 cells/mm^3^, and viral load was 1,310,000/*μ*L. A CT scan of the lungs showed bilateral diffuse alveolar infiltrates (Figure 2).

The patient was admitted and treated empirically with ceftriaxone. The bronchus aspirate and BAL cultures tested positive for *Pneumocystis jirovecii* and *R. equi*, the last confirmed by API identification. Antimicrobial susceptibility testing for *Rhodococcus* was not performed for technical reasons. The strain was sent to an external lab for susceptibility testing, but not recovered there. There was no back-up strain for retesting. An empirical treatment with clindamycin (600 mg three times a day) and clarithromycin (500 mg twice a day) was started together with primaquine to cover *P. jirovecii*. Clinical evolution could not be determined due to loss of follow-up after hospitalization.

### 2.3. Case 3

A 45-year-old man recently diagnosed with HIV infection presented at the emergency department in December 2022 with fever, progressive dyspnea, and right-sided pleuritis-like chest pain, one month after starting HAART. He was severely immunocompromised with a CD4 count of 17/*μ*L. He reported no drug use or recent travel, animal contact, or known TB contact. He was born and raised in Slovenia and worked as a foreman in the harbor of Antwerp. As part of this work, he came into close contact with containers filled with horse hides.

**Figure 3:**
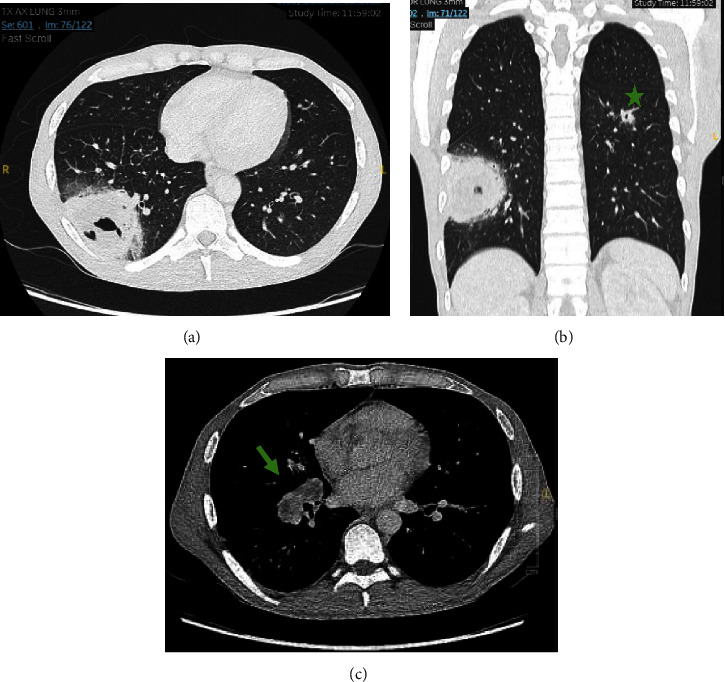
Chest computed tomography of Case 3 at admission: necrotizing cavitating pneumonia in the right lower lobe with adjacent satellite lesions (a) and a smaller cavitating consolidation apically in the left lower lobe ((b), star). Bilateral hilar necrotizing adenopathies ((c), arrow).

Clinical examination showed a cachectic man with a temperature of 38.4°C, a heart rate of 146 beats per minute, a blood pressure of 130/76 mmHg, a saturation of 99% with 1 L of oxygen, and a respiratory rate of 29 per minute. Lung auscultation revealed crepitations on the right side and skin examination diffuse scratch-like lesions. Blood analysis showed a CRP level of 165.5 mg/L (< 10.0 mg/L) and a white blood cell count of 2.39 × 10^9^/L (4.2–10.3 × 10^9^/L). The arterial blood sample revealed respiratory alkalosis (pH 7.540, pCO2 27.3 mmHg). CoV-SARS2, Influenza A and B, and RSV PCR were negative. CT scan of the lungs showed a necrotizing cavitating pneumonia in the right lower lobe, a smaller cavitating consolidation apically in the left lower lobe, and bilateral hilar necrotizing adenopathies (Figure 3). The differential diagnosis included malignancy*, Staphylococcus aureus* infection, TB, or fungal infection. The patient was placed in isolation and empiric antibiotic therapy with intravenous amoxicillin clavulanic acid was initiated.

Bronchoscopy showed a tumor lesion in the right lower lobe. Histological examination of the lesion showed chronic bronchitis with histocyte infiltrate, Ziehl–Neelsen staining and Grocott staining were negative, and there was no evidence of malignancy. Mycobacterial qPCR (RealAccurate Quadruplex Mycobacteria PCR kit; Pathofinder) and auramine staining were negative in BAL fluid, and Aspergillus antigen was 0.16 (normal < 0.50). qPCR for respiratory pathogens (*Chlamydia pneumoniae*, *Legionella pneumophila*, *Streptococcus pneumoniae*, *Mycoplasma pneumoniae*, and *P. jirovecii*) was also negative. Cultures of BAL and an endotracheal aspirate yielded *Rhodococcus* species (25,000 CFU/mL). *Rhodococcus hoagii/equi* was identified via MALDITOF-MS and confirmed by 16 S sequencing. The samples were negative for fungi and mycobacteria. Empiric antimicrobial treatment for *Rhodococcus* spp. was started with azithromycin (250 mg once a day) and rifampicin (600 mg once a day). HAART was switched because of interactions with rifampicin. The patient improved clinically and was discharged from the hospital after one week.

Drug susceptibility testing, performed according to CLSI M24 protocol [8], showed susceptibility to clarithromycin (MIC 0.125 mg/L) and rifampicin (MIC 0.125 mg/L).

At follow-up visits, he reported steady improvement, and after 9 weeks of therapy, rifampicin was stopped and azithromycin was continued for four more weeks.

**Figure 4:**
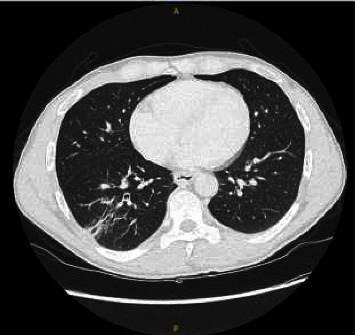
Chest computed tomography of Case 3 four months after starting treatment.

Four months after hospital discharge, his clinical condition continued to improve. A new CT scan showed a decrease in the volume of the cavitary lung lesions and lymphadenopathies (Figure 4), the CD4 count had normalized, and the HIV viral load had remained undetectable. He had returned to work full-time and his exertion tolerance had normalized.

## 3. Discussion

We describe three cases of human *Rhodococcus* infection in patients infected with HIV. All three presented with pneumonia. Long-term follow-up could not be determined for all of them, but as far as we know, none of them died.

Although it is a rare infection, the incidence of *R. equi* increased in the era of the HIV epidemic (pre-HAART era) and because of advances in cancer treatment and organ transplantation, leading to an increase in the number and survival of immunocompromised patients [4, 9]. In addition, the identification techniques improved, leading to an increased detection rate [2, 4]. Our first case dates from 2010 and we possibly, as discussed below, have underestimated the true incidence.

Although *R. equi* is often considered an opportunistic pathogen, infections are, although less frequently, reported in immunocompetent patients (around 10%–15% of all reported cases) [10]. Mortality in this population of patients is approximately 11% and significantly lower compared to patients with (pre-HAART) HIV and other related immune suppression (respectively 50%–55% and 20%–25%) [11]. In addition to immune suppression, other possible predictors of mortality are inappropriate antibiotic treatment and extensive pulmonary disease [12]. Survival in HIV-infected patients improved significantly in the HAART era with a current mortality rate of 8% [12].

About one-third of infected humans have proven contact with herbivores, in particular foals, or their manure [4]. The predominant route of transmission is the inhalation of infected dust particles and aerosols, but direct percutaneous inoculation and hematogenous dissemination have been described [11]. Two out of our three cases were exposed to the farming/livestock environment (exposure to goats in Case 2 and to horse hides in Case 3). Only a very limited number of cases of person-to-person and nosocomial transmission have been reported [11].

Pulmonary involvement and, in particular, cavitary pneumonia (> 80% of cases with pulmonary infection) are present in more than 95% of immunocompromised patients [12]. *R. equi* has a predilection for the upper lobes [9, 11, 12]. All our patients presented cavitary pneumonia in the setting of decreased cell-mediated immunity. Extrapulmonary manifestations such as brain abscesses, lymphadenitis, skin and soft tissue infections, osteomyelitis, endophthalmitis, and bacteremia have been described [9, 11]. Previous studies have found that extrapulmonary infections occur more frequently in immunocompetent patients [12].

The clinical presentation of pulmonary disease consists of a combination of nonspecific symptoms (fever, fatigue, cough, and pleuritic chest pain) and subacute onset. Radiographic findings are difficult to distinguish from other causes of cavitary pneumonia such as TB [11]. Therefore, a definitive diagnosis requires the identification of the causative pathogen either by culturing sputum, BAL fluid, or any other body fluid or tissue when applicable, or by molecular diagnostics (e.g., specific PCR). Special caution is needed because Gram stain of *Rhodococcus* spp. shows pleomorphic Gram-positive coccobacilli that could suggest normal respiratory flora [7]. This highlights the importance of additional deep-seated BAL fluid analysis to facilitate diagnosis. Often colonies grow after 48–72 h of incubation, and this can contribute to a delayed or misleading result due to possible overgrowth in mixed cultures [13]. Additionally, *Rhodococcus* spp. are highly variable in colony morphology, appearing rough, smooth, or mucoid, and different colors are possible, making it hard to identify based on morphology. Moreover, being partially acid-fast, *Rhodococcus* spp. can be mistaken for *Mycobacterium*. Cultures and molecular diagnosis are needed to differentiate between these two infections or establish co-infection [4]. Typical histopathological findings are necrotic tissue containing inflammatory changes of histiocytes with eosinophilic granular cytoplasm [7].

Until today, no randomized controlled trial (RCT) has been performed on the optimal treatment of *Rhodococcus* spp. infections and, more specifically, pneumonia. Most treatment guidelines are based on in vitro evaluations of antimicrobial susceptibility and expert opinion, based on case studies [12]. The antimicrobial susceptibility of *R. equi* is variable and resistance is increasing, making drug susceptibility testing crucial in the optimal management of these infections [12]. In the United States, a “screen and treat” method of foals for *R. equi* has been implemented in a number of farms, possibly leading to the emergence of multidrug resistance [14]. One study found that “screen and treat” was associated with a 40% increase in the prevalence of combined macrolide–rifampicin resistance [15]. According to the One Health principle, a strong case can be made to restrict antibiotic use in foal farms to symptomatic animals. This could preserve the efficacy of antimicrobials for other animals, humans, and the broader environment [14].

In humans, a standard antimicrobial susceptibility testing panel for *R. equi* is not yet available [12]. CLSI recommends a broth microdilution for the susceptibility testing of aerobic actinomycetes. Although gradient diffusion tests have been reported, the comparability of this method with the broth microdilution method is under discussion [16]. CLSI also advises against disk diffusion testing for this organism group [16]. *The* susceptibility of *R. equi* to vancomycin is almost 100% [7]. Susceptibility to carbapenems, macrolides, rifamycins, and fluoroquinolones varies but is still over 90% in certain regions [7]. Other possibly active antimicrobials are tetracyclins, trimethoprim–sulfamethoxazole, and clindamycin [7]. Direct contact with the microbiology department is essential for susceptibility testing, as timely and adequate treatment is important for the prognosis. Besides susceptibility testing, a multiplex qPCR for the detection of related macrolide and rifampicin resistance genes is available [17].

Due to its variable susceptibility pattern and high propensity to develop antimicrobial resistance during treatment, a combination treatment of minimal two antimicrobials is recommended [12, 18]. Treatment should consist of at least one antimicrobial with intracellular activity such as a macrolide or rifamycin. In case of severe disease, a bactericidal antimicrobial such as a glycopeptide or carbapenem should be added to decrease the bacterial load [7, 12]. Antimicrobial resistance development during treatment with rifampin monotherapy, erythromycin, and trimethoprim–sulfamethoxazole has been observed [7, 12]. Drug–drug interactions between antirhodococcal antimicrobials (such as rifampicin) and antiretroviral drugs (and other) must be taken into account and can complicate the treatment of these infections. For example, the elimination of a number of antiretrovirals is accelerated by rifampin, necessitating treatment adjustments—as illustrated in our last case.

Some authors suggest a 10–14 day treatment with active antimicrobial drugs, similar to the treatment of other difficult-to-treat bacterial pneumonia. Treatment duration should, however, depend on several factors, such as the type of infection, the degree of tissue involvement (for example, abscesses and necrotic lesions), and the underlying immune status of the host. Treatment of underlying immunosuppressive conditions and, in some cases, surgery improve the prognosis of *R. equi* infection [12]. Prolonged treatment (months to years), as recommended until recently, no longer seems necessary, unless the infection is not cleared well due to persistent compromised immunity [7, 12]. Clinical and radiological follow-up is necessary to determine the optimal duration of treatment. Currently, there is no role for primary or secondary prophylaxis [9, 11].

In conclusion, we present three cases of *R. equi* pneumonia, an uncommon but severe infection in patients with advanced HIV infection. Clinicians should consider *R. equi* in an immunosuppressed patient with cavitating pneumonia. Diagnosis may be delayed due to nonspecific clinical presentation and certain challenges in the microbiological identification process. Rapid and adequate treatment remains a challenge. Future research on epidemiology and optimal treatment regimens and duration would be useful.

## Data Availability

Anonymized patient-related data and microbiology data can be obtained by following email: ctheunissen@itg.be. Detailed information about the primers and probes used for the respiratory PCR can be obtained by following email: sarah.vandamme@uza.be.
